# Immune Defenses of the Invasive Apple Snail *Pomacea canaliculata* (Caenogastropoda, Ampullariidae): Phagocytic Hemocytes in the Circulation and the Kidney

**DOI:** 10.1371/journal.pone.0123964

**Published:** 2015-04-20

**Authors:** Juan A. Cueto, Cristian Rodriguez, Israel A. Vega, Alfredo Castro-Vazquez

**Affiliations:** 1 Instituto de Fisiología, Facultad de Ciencias Médicas, Universidad Nacional de Cuyo, Mendoza, Argentina; 2 Instituto de Histología y Embriología “Dr. Mario H. Burgos”, Consejo Nacional de Investigaciones Científicas y Técnicas, Mendoza, Argentina; 3 Área de Biología, Facultad de Ciencias Exactas y Naturales, Universidad Nacional de Cuyo, Mendoza, Argentina; Uppsala University, SWEDEN

## Abstract

Hemocytes in the circulation and kidney islets, as well as their phagocytic responses to microorganisms and fluorescent beads, have been studied in *Pomacea canaliculata*, using flow cytometry, light microscopy (including confocal laser scanning microscopy) and transmission electron microscopy (TEM). Three circulating hemocyte types (hyalinocytes, agranulocytes and granulocytes) were distinguished by phase contrast microscopy of living cells and after light and electron microscopy of fixed material. Also, three different populations of circulating hemocytes were separated by flow cytometry, which corresponded to the three hemocyte types. Hyalinocytes showed a low nucleus/cytoplasm ratio, and no apparent granules in stained material, but showed granules of moderate electron density under TEM (L granules) and at least some L granules appear acidic when labeled with LysoTracker Red. Both phagocytic and non-phagocytic hyalinocytes lose most (if not all) L granules when exposed to microorganisms *in vitro*. The phagosomes formed differed whether hyalinocytes were exposed to yeasts or to Gram positive or Gram negative bacteria. Agranulocytes showed a large nucleus/cytoplasm ratio and few or no granules. Granulocytes showed a low nucleus/cytoplasm ratio and numerous eosinophilic granules after staining. These granules are electron dense and rod-shaped under TEM (R granules). Granulocytes may show merging of R granules into gigantic ones, particularly when exposed to microorganisms. Fluorescent bead exposure of sorted hemocytes showed phagocytic activity in hyalinocytes, agranulocytes and granulocytes, but the phagocytic index was significantly higher in hyalinocytes.

Extensive hemocyte aggregates ('islets') occupy most renal hemocoelic spaces and hyalinocyte-like cells are the most frequent component in them. Presumptive glycogen deposits were observed in most hyalinocytes in renal islets (they also occur in the circulation but less frequently) and may mean that hyalinocytes participate in the storage and circulation of this compound. Injection of microorganisms in the foot results in phagocytosis by hemocytes in the islets, and the different phagosomes formed are similar to those in circulating hyalinocytes. Dispersed hemocytes were obtained after kidney collagenase digestion and cell sorting, and they were able to phagocytize fluorescent beads. A role for the kidney as an immune barrier is proposed for this snail.

## Introduction

Haemocytes are the cellular component of the molluscan immune system and their functions include recognition and phagocytosis of invaders [1–3] or capsule formation around larger foreign objects [4–6]. Besides immunity, hemocytes serve a variety of functions such as blood hemostasis and wound healing [7], shell formation and repair [7, 8], nutrient digestion and excretion [9]. Hemocytes also have been involved in the stress response through releasing vertebrate like endocrine molecules [10, 11].

Morphological characterization of gastropod hemocytes have been made mainly in heterobranchs (e.g., [12, 13–17]) but there is some information on other gastropods, including *Pomacea canaliculata* [18–20]. A diversity of cell types has been described, but there is a growing confusion caused by different terminologies developed by different authors in a growing number of species. Table 1 is an attempt to synonymize the information on hemocytes of architaenioglossan gastropods (which include ampullariids) on the basis of the simple terminology used by Cheng [16] and which is largely based on the work of early authors (cited by [21]): hyalinocytes, agranulocytes and granulocytes).

**Table 1:** Hemocyte types according to Cheng [16] and proposed synonymy in architaenioglossan gastropods (families Viviparidae and Ampullariidae).

Species (Family)	Hyalinocytes	Agranulocytes	Granulocytes	Methodology	Reference
*Bellamya bengalensis* (Viviparidae)	Semigranulocytes	Agranulocytes	Granulocytes	Flow cytometry, phase contrast, Giemsa stain	[22]
*Viviparus* sp. (Viviparidae)	Large hyaline amoebocytes	Small hyaline amoebocytes	Large amoebocytes with eosinophilous granules	Not mentioned	Kollmann (1908), cited in [21], p. 279
*Viviparus ater* (Viviparidae)	No types were distinguished	No types were distinguished	No types were distinguished	Flow cytometry, May-Grünwald-Giemsa stain	[23]
*Viviparus ater* (Viviparidae)	No types were distinguished	No types were distinguished	No types were distinguished	Transmission electron microscopy, acid phosphatase reactivity	[24]
*Pila globosa* (Ampullariidae)	Granulocytes I (= progranulocytes) Granulocytes II	Agranulocytes = hyalinocytes	Granulocytes III	May-Grünwald-Giemsa stain	[14]
*Pila globosa* (Ampullariidae)	Semigranulocytes	Agranulocytes	Granulocytes	Phase contrast, Giemsa stain	[22]
*Pomacea canaliculata* (Ampullariidae)	Cells with electron lucid granules	Nongranular cells	Cells with electron dense granules	Transmission electron microcopy	[18]
*Pomacea canaliculata* (Ampullariidae)	Hyalinocytes	Agranulocytes	Granulocytes	May-Grünwald-Giemsa stain	[20]
*Pomacea canaliculata* (Ampullariidae)	Group II, “agranular cells”	Group I, “blast-like cells”	Goup II, “granular cells”	Flow cytometry, May-Grünwald-Giemsa stain, transmission electron microscopy	[19]

Particularly in *P*. *canaliculata* (an invasive species, [25]) there is some evidence for the existence of these three hemocyte types [18–20]. However, sorting of hemocyte populations has only been partially successful in the past [19] and the attempts to show the hemocyte ultrastructure were insufficient because of dark micrographs [18] or a magnification insufficient to show all hemocyte granules and organelles [19]. Also, the differential phagocytizing ability of hemocytes as well as the changes in hemocyte granules and other acidic compartments after microbial exposure have not been studied.

Also, hemocyte islets have been mentioned in the kidney of *P*. *canaliculata* [26, 27], a beret-shaped epithelial organ that covers the coiled intestine and dorsally delimits the renal chamber [28, 29]. Because of its position in the circulation, the kidney filters hemolymph from the head-foot mass and from part of the visceral hump [28] and hence, hemocytes therein may constitute an important immune barrier and they may serve the role of a hematopoietic organ. Studies of hematopoiesis in gastropods [30] are scarce if compared with other groups, notably fruit-flies [31] and crustaceans [32–34].

The current paper was aimed to characterize the hemocytes found in the circulation and renal islets in *P*. *canaliculata* under both light and electron microscopy, as well as their changes after microbial (yeasts and bacteria) or fluorescent bead exposure. Also, separation of circulating hemocyte populations corresponding to the three cell types have been successfully achieved using flow cytometry and cell sorting and the differential ability of the sorted hemocytes to phagocytize fluorescent beads has been shown. Furthermore, the phagocytizing ability of renal hemocytes has been shown *in vivo* and they have been separated from renal epithelial cells by flow cytometry, and their phagocytizing ability has also been shown *in vitro*.

## Materials and Methods

### Animals and culturing conditions

Adult males obtained from a cultured strain of *P*. *canaliculata* were used. Collection of the original stock did not require specific permits since it is not an endangered but rather an invasive species (IUCN Red List status "Least concern"; population trend: "increasing"). The original stock was collected at the Rosedal Lake, 34°34’ S; 58°25’ W, Palermo, Buenos Aires, Argentina. Palermo is the neotype locality of *P*. *canaliculata* [35]. Voucher (ethanol preserved) specimens of the original population, were deposited at the collection of Museo Argentino de Ciencias Naturales (Buenos Aires, Argentina; lots MACN-In 35707 and MACN-In 36046, respectively). Temperature was regulated at 24–26°C and artificial lighting was provided 14 h per day. Aquarium water was changed thrice a week. Animals were fed ad libitum with a mixed diet made of fresh lettuce, supplemented weekly with carp food pellets (Peishe Car Shulet, Argentina), desiccated and powdered *P*. *canaliculata* eggs and toilet paper (Higienol, Argentina).

### Hemolymph collection

Hemolymph (0.5–1.5 mL per snail) was withdrawn from the ventricle as previously described [20, 36] using a syringe soaked, unless otherwise indicated, with an antiaggregant buffered solution (*Pc*ABS) designed to match normal plasma osmolality and pH of *P*. *canaliculata* (43mM NaCl, 1.8 mM KCl, 10 mM HEPES and 30 mM EDTA; pH 7.6) according to Cueto *et al*. [36].

### Circulating hemocyte populations: morphology and phagocytosis

#### HE stain and phase contrast microscopy

A drop of hemolymph (50 μL) was allowed to settle for 10 min onto a glass slide, to permit hemocyte attachment. Afterwards, hemocytes were fixed by adding 100 μL of Bouin’s fluid for 30 min at room temperature (2 replicates of samples obtained from each of 6 snails). Then the slides were gently washed in *Pc*ABS and were stained with either Harris hematoxylin-eosin (HE stain). This fixation and staining procedure gave better results than the traditional Romanowsky-type stains, particularly for showing the eosinophily of the granules. Slides were mounted with Eukitt and a coverslip. Hemocyte morphology was examined and photographed under a Nikon Eclipse 80i Microscope using Nikon DS-Fi1-U3 camera and Nikon NIS-ELEMENT Image Software for image acquisition. Hemocyte counts were made with a Neubauer’s hemocytometer. Alternatively, living cells were observed using phase contrast microscopy, after 10 min attachment.

#### Flow cytometry and cell sorting

Hemolymph was analyzed immediately after withdrawal in a FACS Aria III (BD Bioscience, California, USA) flow cytometer. Dot plots for forward light scatter (FSC) and side light scatter (SSC) were used, indicating differences in cell size and complexity-granularity, respectively; 20,000 events per hemolymph sample were recorded. Data acquisition and further analysis were made using DiVa software (version 6.1.3). Fractions containing hemocytes of different sizes and complexity-granularity were obtained by cell sorting and studied after HE stain or under phase contrast microscopy. Similarly sorted cells were used for experiments involving phagocytosis of fluorescent beads (see below).

#### Confocal laser scanning microscopy of acidic compartments

Fresh hemolymph (200 μL, approximately 6 x10^5^ cells per well) was collected in a syringe soaked with buffered solution without EDTA (*Pc*BS: 43mM NaCl, 1.8 mM KCl, 10 mM HEPES; pH 7.6) was seeded in a 24-well plate (Nunclone surface, Nunc, Denmark) at 28°C. Each well was provided with 500 μL of culture medium, according to Cueto *et al*. [20], and with a bottom glass coverslip to which cells were allowed to attach for 15 min. Hemocytes were then treated with 1 μM LysoTracker Red DND-99 (Invitrogen) for 30 min. Finally, cells were fixed in 3% formaldehyde, treated with Hoechst 33258 for DNA staining, mounted in Mowiol and examined under an Olympus FV1000 confocal laser microscope.

#### Transmission electron microscopy (TEM)

Circulating hemocytes were obtained by hemolymph centrifugation (2000 rpm, 7 min at 4°C) and were resuspended in *Pc*ABS and centrifuged again, and fixed in 500 μL 2.5% glutaraldehyde in *Pc*BS for 180 min. After 3 hours fixation at room temperature, the resulting hemocyte pellet was washed twice in *Pc*BS, and embedded in 1.5% low melting point agarose. The resulting agarose ‘blocks’ were post-fixed in 1% osmium tetroxide overnight and then washed twice in *Pc*BS. Finally, the blocks were dehydrated through a graded ethanol series followed by acetone, and embedded in Spurr resin. Thin sections (50–70 nm) were stained with uranyl acetate and lead citrate and examined in a Zeiss 900 transmission electron microscope.

#### *In vitro* phagocytosis of different microorganisms by circulating hemocytes

Suspensions of Escherichia coli (DH5α strain), *Staphylococcus aureus* (untyped) and *Saccharomyces cerevisiae* (Levex, Argentina) were used. Bacteria were cultured in Luria-Bertani medium in a shaker (300 rpm) at 37°C for 24 hours, and were centrifuged (10,000 g, 10 min) to remove the culture medium, resuspended in sterile distilled water and centrifuged again. Bacterial suspensions were adjusted to ~60 CFU (colony forming units)/mL. Dehydrated yeast cells were suspended in sterile distilled water and adjusted to ~12.6 x 10^4^ cells/mL in a hemocytometer. All microorganisms were heat-inactivated by autoclaving before use.

Hemocyte reactions to microbial exposure were observed by both confocal laser scanning microscopy (LysoTracker Red) and TEM. For LysoTracker Red observations, hemocytes (approximately 6 x 10^5^ cells, contained in 200 μL of fresh hemolymph) were allowed to attach (15 min) and were later exposed to LysoTracker Red for 30 min as described above, before the addition of 10 μL of the *E*. *coli* suspension (~60 CFU/mL). The plates were then centrifuged (10 min, 400 *g*, 4°C) and incubated for 60 additional minutes at 28°C. Finally, cells on coverslips were fixed in 3% formaldehyde, treated with Hoechst 33258 for DNA staining, mounted in Mowiol and examined under an Olympus FV1000 confocal laser microscope. Similarly treated hemocytes, but that were not exposed to *E*. *coli* cells, were used as controls.

For TEM observations, 100 μL of each microbial suspension were mixed with 100 μL of freshly withdrawn hemolymph (about 3 x 10^5^ hemocytes) in an Eppendorf tube, and were incubated for 60 min at 28°C. Cells were then centrifuged and the supernatant was replaced by the fixative (2.5% glutaraldehyde in *Pc*BS). Three hours later, the cells were prepared for TEM observations as described above.

#### *In vitro* phagocytosis of fluorescent beads by sorted and unsorted circulating hemocytes

Hemolymph was withdrawn from the ventricle of 6 snails with a dry syringe and the hemocytes were separated by centrifugation (2000 g, 5 min). The hemocytes were then mixed with 100 μL of a suspension of fluorescent beads (Fluoresbrite Yellow Green Microspheres, 1.00 μm; Polysciences Inc.) in cell-free hemolymph plasma, at a ratio of approximately 10 beads per hemocyte. Afterwards, the mixture of hemocytes and beads was spun down for 1 min and then incubated (28°C, 60 min). Each sample was then diluted with 200 μL of PcABS and cell associated fluorescence was determined in the flow cytometer using an air cooled argon laser, providing an excitation at 488 nm and at 633 nm. Fluorescence emission was collected with 530/30 and 630/22 band-pass filters and 20,000 events were acquired per sample. The percentage of cells containing fluorescent beads was determined in a plot displaying fluorescence (FITC) and cell size (FSC). All data analyses were made using FlowJo software. Samples of the cytometer effluxes were allowed to settle for 10 min on a glass slide and observed under Nikon Eclipse 80i Microscope (phase contrast) using a Nikon DS-Fi1-U3 camera and Nikon NIS-ELEMENT Image Software for image acquisition.

Another set of experiments was performed to establish the phagocytizing ability of three hemocyte fractions separated on the basis of different size (FSC) and complexity-granularity (SSC) and in which either hyalinocytes, agranulocytes or granulocytes predominate (see Results). Each hemocyte fraction was recovered on 50 μL plasma, centrifuged (2000 *g*, 5 min) and resuspended in 100 μL fresh plasma containing fluorescent beads at a 10:1 beads/hemocyte ratio. Hemocytes were briefly spun down and then incubated (28°C, 60 min). Afterwards, incubates were diluted with 200 μL *Pc*ABS and cell associated fluorescence was determined in the flow cytometer. Cell viability was 96–98% (propidium iodide exclusion method) and the data presented under Results were normalized to 100%. Also, samples of the cytometer effluxes were allowed to settle for 10 min onto a glass slide and observed under phase contrast microscopy.

### Tissue hemocytes in kidney islets: morphology and phagocytosis

#### HE stain

Animals were immersed in cold water at 4°C for 20–30 minutes (both for producing relaxation and minimizing pain) and then the shell was cracked to obtain the soft parts. Samples of the kidney (= ‘posterior’ kidney in Andrews, [28]) were fixed in Bouin’s fluid (diluted 1:1 in distilled water) for one week at 5° C, and were later preserved in 70% ethanol until the samples were dehydrated through a graded ethanol series, cleared in xylene and embedded in a 1:1 paraffin-resin mixture (Histoplast, Argentina) and sectioned (5 μm) in a rotary microtome (Microm HM 325), stained with Harris hematoxylin-eosin, and mounted in Eukitt.

#### TEM

Kidney samples were fixed in 4% paraformaldehyde and 2.5% glutaraldehyde in *Pc*BS for 5 hours, post-fixed in 1% osmium tetroxide for 90 min at room temperature, washed, dehydrated through a graded ethanol-acetone series, and embedded in Spurr resin. Thin sections (50–70 nm) were stained with uranyl acetate and lead citrate.

#### *In vivo* microbial phagocytosis by hemocytes in renal islets

Animals were injected in the foot (200 μL) with suspensions of different heat-inactivated microorganisms (*E. coli*, *S. aureus* or *S. cerevisiae*, prepared as describe above) and kidney samples were obtained 2 h after injection and processed for TEM. Sham-injected controls were used.

#### *In vitro* phagocytosis of fluorescent beads by hemocytes obtained from renal islets

Kidney samples (~50 mg) were obtained from 6 snails, cut into small pieces with a razor blade and digested in 600 μL of 0.1% collagenase (Sigma C5138) for 20 min at room temperature. Tissue debris were decanted and 500 μL of the supernatant were centrifuged (2000 *g*, 5 min) and made up to 1,500 μL with *Pc*ABS. The cell suspensions were a mixture of hemocytes, renal cells and urinary concretions ([37] and this paper) and were further studied by flow-cytometry. The samples were excited with an argon ion laser at 488 nm and the instrument was set to capture the fluorescence signals at 520 nm (PI-A) and a scatter plot of size (FSC-A) and internal complexity (SSC-A) was obtained (20,000 events per sample). In each case, a region in which hemocytes predominate was framed on a FSC versus PI plot and was sorted and exposed to fluorescent beads. The phagocytic index was determined as described above for circulating hemocytes. Cell viability in parallel experiments was 58–63% (propidium iodide exclusion method) and the data presented under Results were normalized to 100%.

## Results

### Morphology and phagocytosis reactions of circulating hemocytes

#### HE stain and phase contrast microscopy of circulating hemocytes and of hemocyte populations sorted by flow cytometry

**Fig 1:**
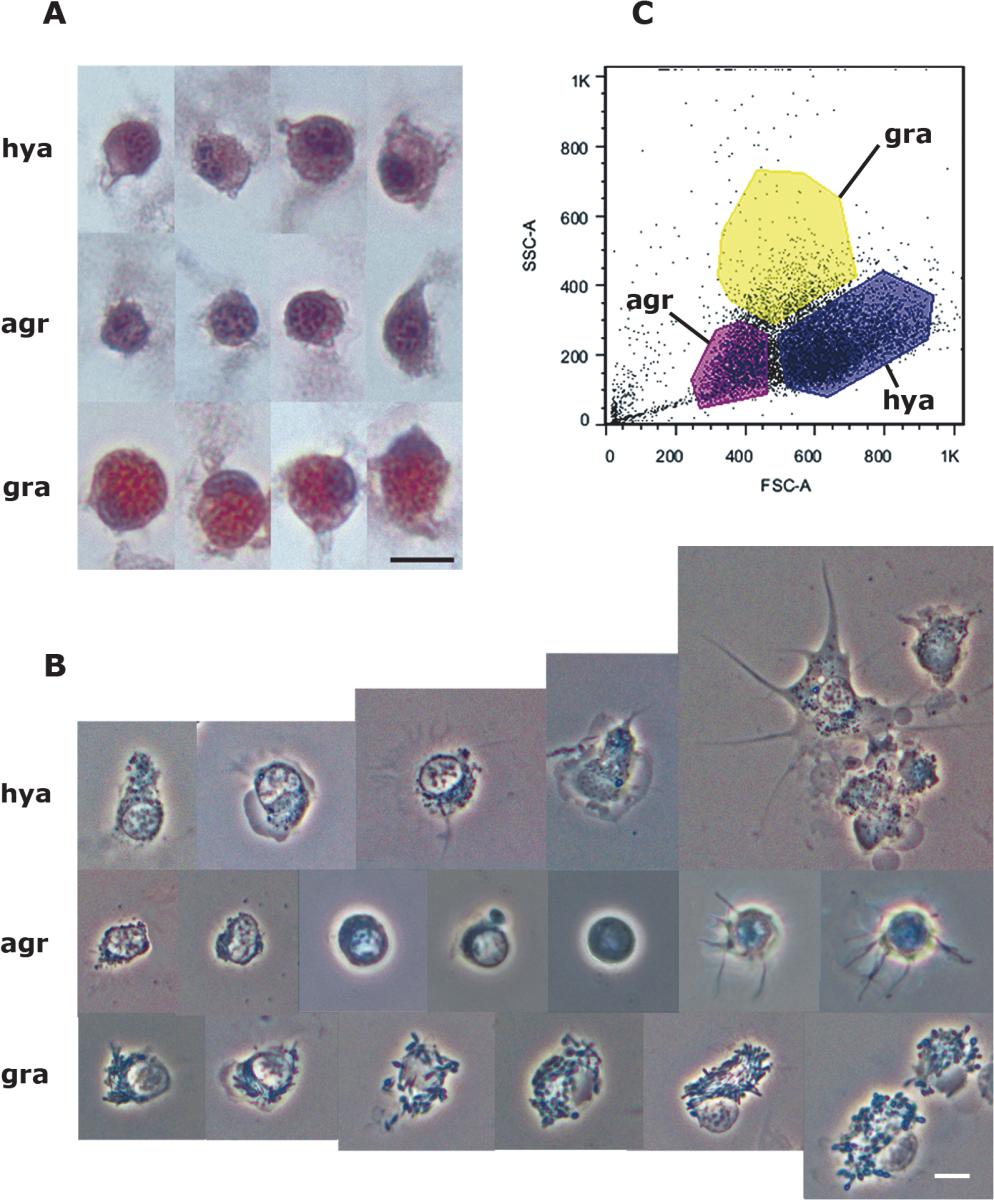
Circulating hemocyte types and their separation by flow cytometry. **(A)** Examples of hyalinocytes, agranulocytes and granulocytes in hemolymph smears (HE-stain). **(B)** Examples of living hyalinocytes, agranulocytes and granulocytes attached onto a glass slide (phase contrast). **(C)** Flow cytometry (dot plot of size *vs*. complexity-granularity) of a representative hemolymph sample indicating the three areas that were chosen for cell sorting, where the three hemocyte types were predominant. Abbreviations: *agr*, agranulocytes; *gra*, granulocytes; *hya*, hyalinocytes. Scale bars represent 5 μm.

Representative examples of the three hemocyte types (hyalinocytes, agranulocytes and granulocytes) are shown in Fig 1A and 1B (HE stain and phase contrast, respectively).

HE stained hyalinocytes lack cytoplasmic granules and are the most abundant in the circulation (63.0± 3.8%); they show an extended basophilic or chromophobic cytoplasm and an eccentric nucleus of variable shape (either round, bean-shaped or bilobed). Agranulocytes (28.1 ± 1.6% of circulating cells) also lack granules under light microscopy and are characterized by a round nucleus and a scarce cytoplasm. Granulocytes represent a small proportion of cells in the circulation (8.9 ± 2.6%). They are large cells showing an eccentrically located nucleus of variable shape and their cytoplasm is loaded with large eosinophilic granules.

Correlative findings were obtained with phase contrast microscopy of living hemocytes (Fig 1C). Living cells were generally larger than the fixed ones, and the nuclei were always rounded. Granulocytes showed their characteristic large granules but both hyalinocytes and agranulocytes showed some ‘granularity’ which may be correlated with other granules and organelles seen under TEM (see below). Hyalinocytes were seen emitting lamellipodia and filopodia, while some agranulocytes were only emitting filopodia. No circulating granulocytes appeared emitting either lamellipodia or filopodia.

Flow cytometry was performed on freshly withdrawn hemolymph samples and three regions were delimited on the basis of different cell size and complexity- granularity by cell sorting, and predominantly contained hyalinocytes, agranulocytes or granulocytes (regions *hya*, *agr* and *gra*, respectively; Fig 1C).

#### Hemocyte acidic compartments

**Fig 2:**
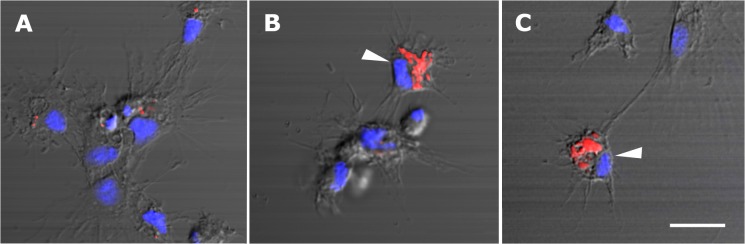
Circulating hemocytes (LysoTracker Red-Hoechst 33258). **(A)** A group of spreading hyalinocytes; small acidic granules (red) are seen in some of them. **(B)** A spreading granulocyte (arrowhead) showing numerous rod-shaped, acidic granules; also, there is a group of spreading and round hyalinocytes, which are essentially devoid of acidic granules. **(C)** Another spreading granulocyte (arrowhead) showing large and merging acidic granules; also, there are two spreading hyalinocytes with no acidic granules. Scale bar represents 10 μm.

Most cells appeared spread and attached onto the substrate and two distinct types of acidic granules were observed in them: small round granules which did not displace the nucleus (Fig 2A) and large elongated granules which were numerous and displaced the nucleus to an eccentric position (Fig 2B). Because of their size and distribution in the cytoplasm, the latter granules should correspond to the eosinophilic granules seen with HE staining, the large granules seen under phase contrast microscopy, and the R granules observed in granulocytes under the electron microscope (see next section). These granules were also merging into even larger granules in some cells (Fig 2C) and the same was observed under phase contrast (not shown). The small round acidic granules may correspond to L granules observed in hyalinocytes under the electron microscope (next section) and may account for part of the ‘granularity’ observed under phase contrast microscopy.

#### Ultrastructure of circulating hemocytes

**Fig 3:**
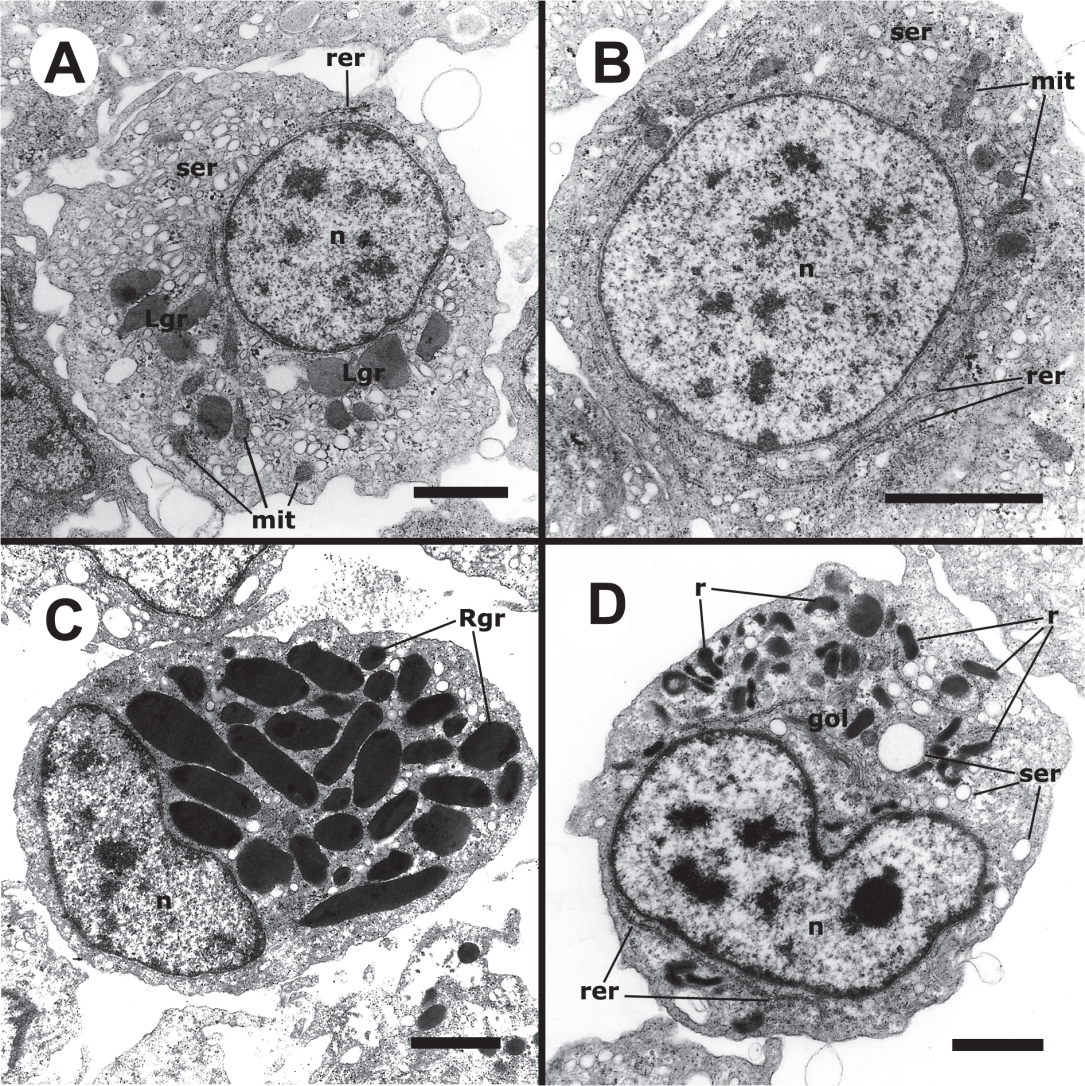
Circulating hemocytes (TEM). **(A)** Hyalinocyte with an eccentric nucleus, numerous SER vesicles, mitochondria and L granules; a few profiles of the RER are also seen. **(B)** Agranulocyte with a round central nucleus, SER vesicles and RER cisternae, as well as some mitochondria. **(C)** Granulocyte, showing a displaced, bean-shaped nucleus and numerous R granules; some SER vesicles are also seen. **(D)** Pro-granulocyte, showing a bean-shaped nucleus, a Golgi complex and numerous immature R granules of varying electron density; some SER vesicles and extended RER cisternae are also seen. Abbreviations: *gol*, Golgi complex; *Lgr*, L granules; *mit*, mitochondria; *n*, nucleus; r, immature R granules; *Rgr*, mature R granules; *rer*, RER cisternae; *ser*, SER vesicles. Scale bars represent 1 μm.

**Fig 4:**
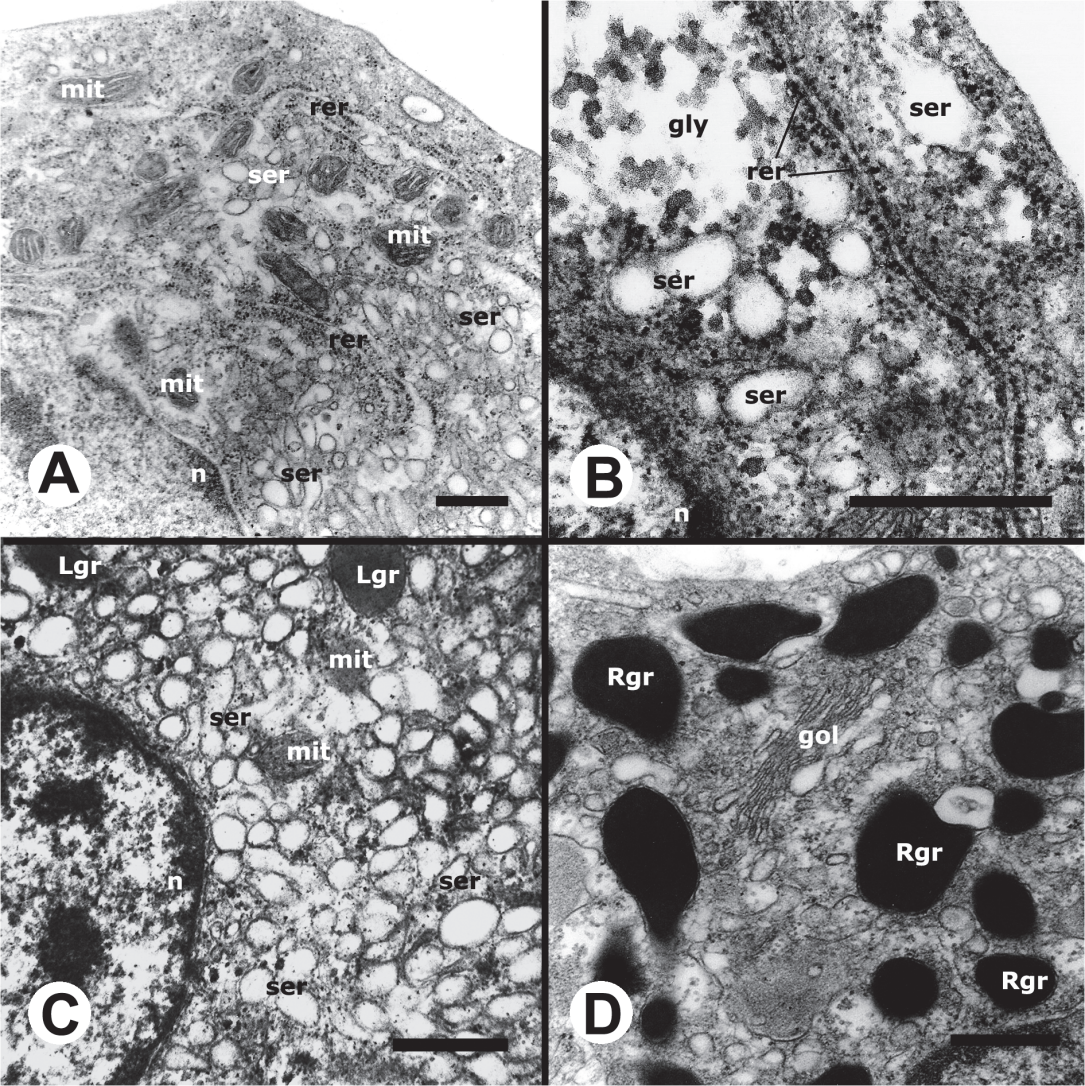
Details of circulating hemocytes (TEM). **(A)** Cytoplasm of a hyalinocyte showing numerous mitochondria and some RER and SER profiles. **(B)** Detail of another hyalinocyte showing an extended RER cisterna, SER vesicles and a membrane-unbound zone with presumptive glycogen granules. **(C)** Cytoplasm of a hyalinocyte showing numerous SER vesicles, as well as a few L granules and mitochondria. **(D)** Cytoplasm of a granulocyte showing numerous R granules around a Golgi complex. Abbreviations: *gly*, presumptive glycogen granules; other abbreviations as in Fig 3. Scale bars represent 1 μm.

A well developed smooth endoplasmic reticulum (SER) and a varying number of mitochondria (many of them elongated) were common features of all hemocytes but were more evident in hyalinocytes because of their large cytoplasm and scarcity of granules (Figs 3 and 4). Hyalinocytes (Figs 3A and 4A–4C) also showed an eccentrically located nucleus and flattened and extended cisterns of the rough endoplasmic reticulum (RER), usually in the vicinity of the nucleus. Free ribosomes were frequently seen. Membrane-bound granules of moderate electron density (not recognized in HE stained preparations) were found in most hyalinocytes; they will be referred to as L granules, because of their similarity to lysosomes. The SER was formed by numerous round or oval small vesicles and sometimes by larger vacuoles. The Golgi apparatus was only infrequently found. Also, presumptive glycogen stores (not membrane-bound) were also occasionally seen (Fig 4B).

Agranulocytes (Fig 3B) exhibited a round nucleus and a narrow cytoplasmic band around it. Otherwise, their cytoplasmic ultrastructural features were similar to those in hyalinocytes except that L granules were infrequent.

The large cytoplasm of mature granulocytes could be easily recognized under TEM, because of the numerous electron-dense granules, which were membrane-bound and elongated (Fig 3C) and may correspond to the eosinophilic granules seen in HE stained preparations, and to the large granules appearing under phase contrast microscopy. The nuclei of these cells varied in shape and were always eccentrically located. Early stages of granulocyte differentiation were characterized by a round nucleus and by small granules of varying electron density (Fig 3D), which may later coalesce into larger, rod-like ones. The Golgi complex was well developed in both mature granulocytes and in early granulocyte stages (Figs 3D and 4D). Also, occasional L granules as those of hyalinocytes were found in mature circulating granulocytes (not shown in Figs 3 and 4).

#### Changes of acidic compartments after in vitro exposure of circulating hemocytes to bacteria

**Fig 5:**
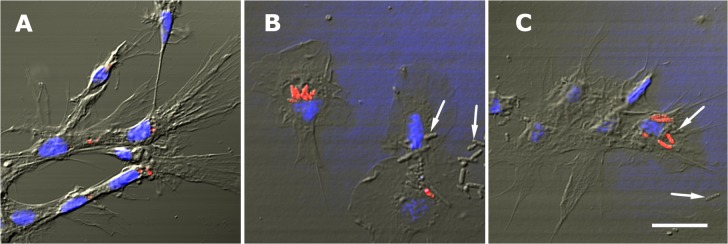
*In vitro* phagocytosis of *E*. *coli* cells by circulating hemocytes (LysoTracker Red-Hoechst 33258). **(A)** A group of control hyalinocytes, some of them showing small acidic granules. **(B)** Hemocytes exposed to *E*. *coli*; a phagocyte (upper left) showing a group of internalized red-labeled bacteria, while another phagocyte (lower right) shows a single internalized bacterium. Small acidic granules are not seen in these hemocytes, whether phagocytic or not. Bacteria which are free over and around hemocytes are not labeled (arrows). **(C)** A group of hemocytes, one of them showing several internalized bacteria in different degrees of digestion. Small acidic granules are not seen in these hyalinocytes. Non internalized bacteria are not labeled by LysoTracker Red (arrows). Scale bar represents 10 μm.

Most control hemocytes (i.e., not exposed to bacteria) showed small and round acidic granules (corresponding to L granules, Fig 5A), while cells bearing rod-like granules (corresponding to R granules) were occasionally seen (not shown in Fig 5) and they were not phagocytizing cells. Hemocytes exposed to an *E*. *coli* suspension behaved differently whether they had internalized bacteria or not: those without internalized bacteria had lost all acidic granules while those with internalized bacteria showed intensely labeled phagocytic vesicles with the shape of the phagocytized bacterium in different stages of digestion (Fig 5B and 5C). Also, unlabeled vesicles sometimes surround the labeled vesicles (Fig 5B), which may be correlated with the phagosomes with more than one compartment that are seen under TEM (see next section). No additional acidic compartments (such as those corresponding to L granules seen in control hemocytes, Fig 5A) are seen in phagocytic hemocytes, which correlates well with the lack of granules observed in phagocytic hemocytes under TEM (next section). Most non-phagocytic hemocytes also appeared devoid of acidic compartments in preparations exposed to bacteria.

#### *In vitro* microbial phagocytosis by circulating hemocytes under TEM

**Fig 6:**
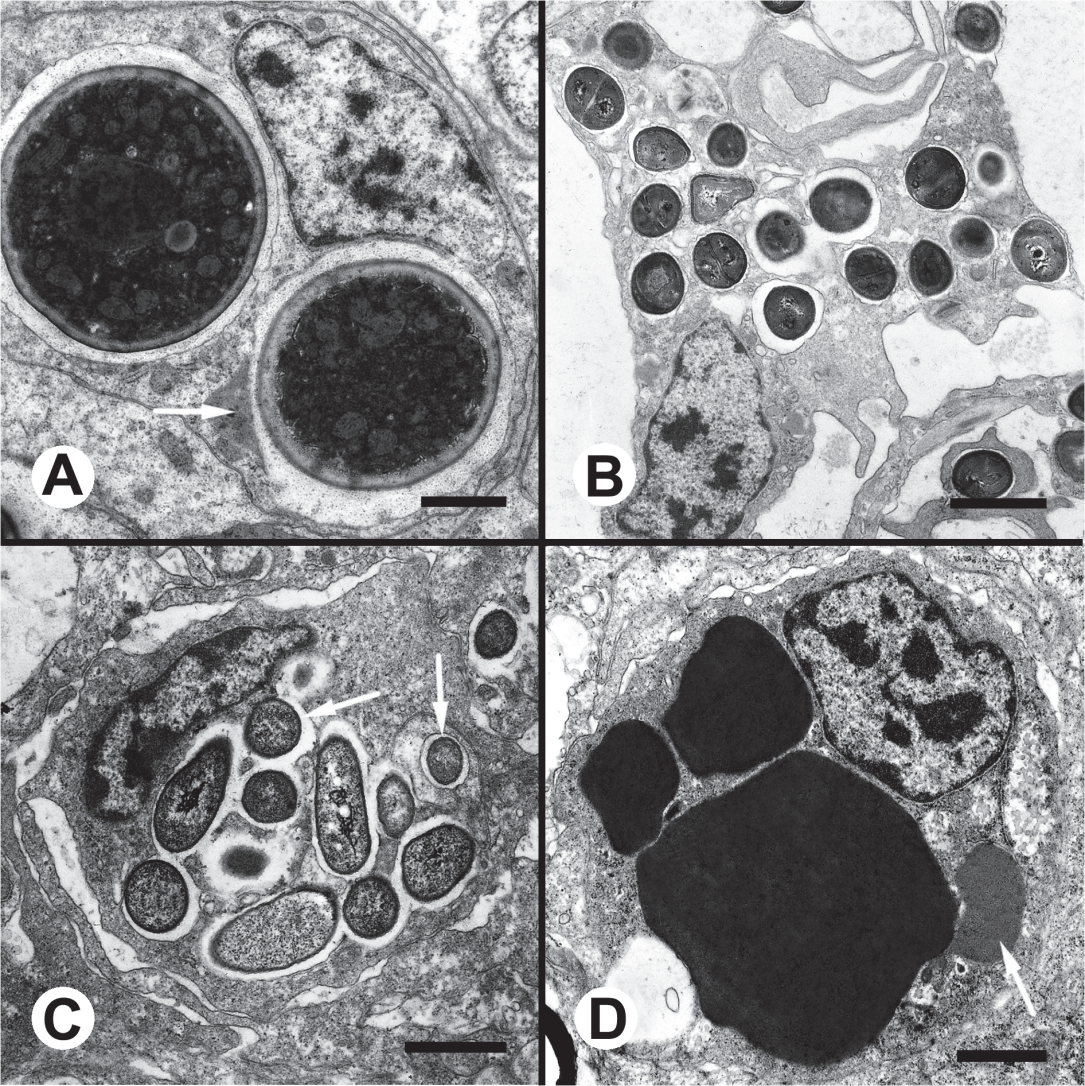
*In vitro* microbial phagocytosis by circulating hemocytes (TEM). **(A)** Two yeast cells engulfed by a phagocytic hemocyte; a large L granule is attached to one of the phagosomes and may be preceding fusion (arrow). **(B)** Numerous *S*. *aureus* cells engulfed by a hemocyte within seldom interconnected phagosomes. **(C)** *E*. *coli* cells may also be phagocytized in large numbers within complex phagosomes which frequently show more than one compartment (arrows). **(D)** Granulocyte in a preparation exposed to *E*. *coli* cells showing extensive R granule fusion and a single L granule.

Hemocytes were able to phagocytize yeast cells in spite of their large size, resulting in a marked distortion of the phagocyte. A microgranular material of low electron density fills the phagosome space surrounding the yeast cell (Fig 6A). Membrane-bound L granules, which were a common feature of unexposed hyalinocytes, were only rarely seen in phagocytic cells, which would indicate that phagocytosis is accompanied either by degranulation (exocytosis) or by fusion of L granules to phagosomes (Fig 6A). Phagocytic hemocytes are most likely to be hyalinocytes, but granulocytes may also be phagocytic (see below) though they may not be recognizable after loss of their characteristic R granules.

*S*. *aureus* cells were internalized in individual phagosomes that were seldom interconnected (Fig 6B). In turn, *E*. *coli* cells were internalized in large phagosomes with multiple, frequently interconnected compartments (Fig 6C, arrows). No phagocytic cells showed R granules, but some granulocytes showed extensive granular fusion with the resulting formation of gigantic R granules (Fig 6D).

#### *In vitro* phagocytosis of fluorescent beads by sorted and unsorted circulating hemocytes

**Fig 7:**
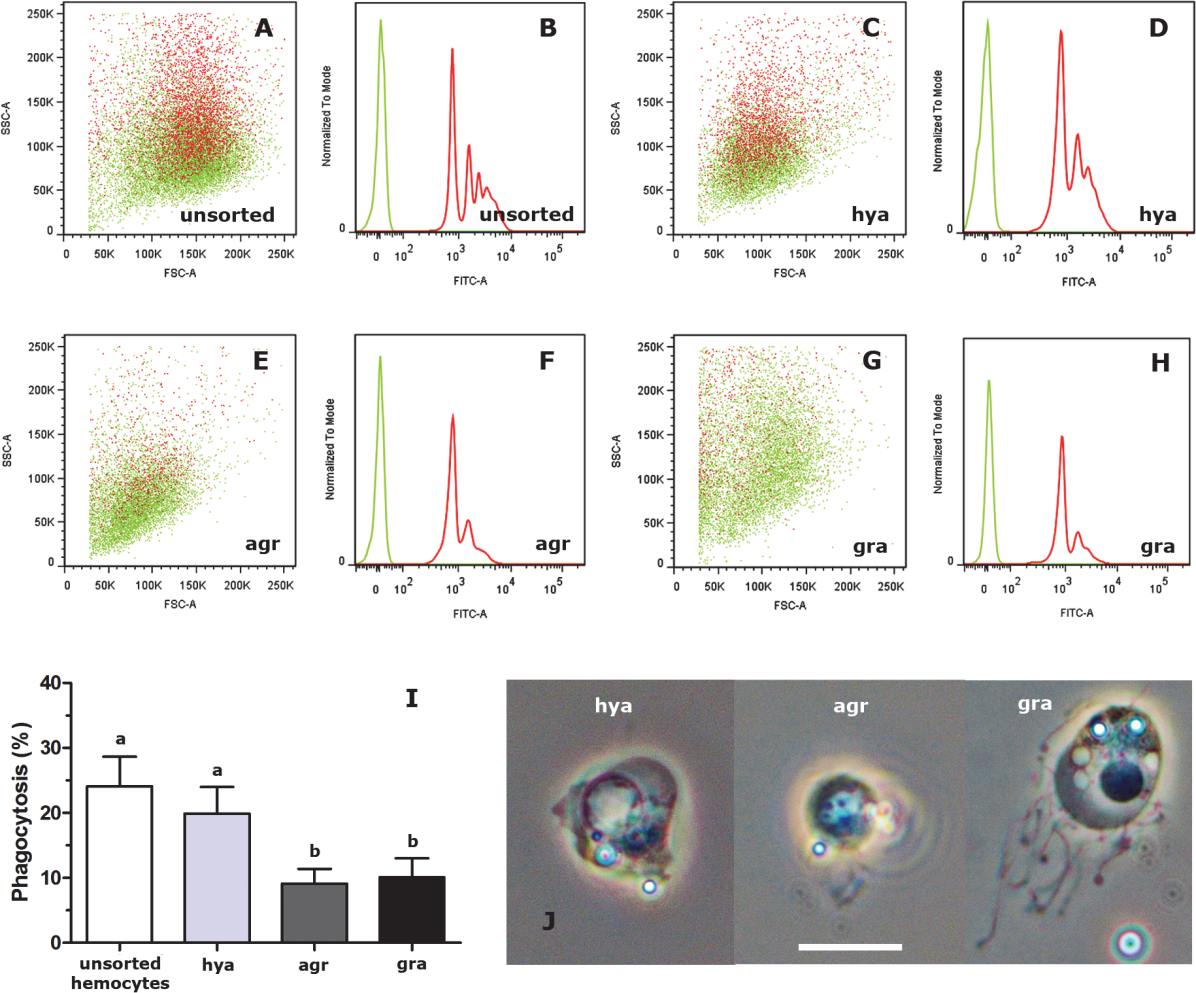
*In vitro* phagocytosis of fluorescent beads by sorted and unsorted circulating hemocytes. **(A)** Dot plot of cell size *vs*. complexity-granularity of unsorted circulating hemocytes exposed to fluorescent beads. In this and in panels C, E and G, red dots indicate phagocytic hemocytes associated to fluorescent beads, while green dots indicate non phagocytic ones. **(B)** Histograms of the sample shown in A: in this and in panels D, F and H, red and green lines show the distribution of phagocytic and non-phagocytic hemocytes, respectively. The red histogram indicates the existence of 4–5 hemocyte populations associated to different amounts of fluorescent beads. **(C)** Dot plot of size *vs*. complexity-granularity of sorted hyalinocytes exposed to fluorescent beads. **(D)** Histograms of the hyalinocyte sample shown in C: the red histogram indicates the existence of 3–4 hyalinocytes populations associated to different amounts of fluorescent beads. **(E)** Dot plot of size *vs*. complexity-granularity of sorted agranulocytes exposed to fluorescent beads. **(F)** Histograms of the agranulocyte sample shown in E: the red histogram indicates the existence of 2–3 agranulocyte populations associated to different amounts of fluorescent beads. **(G)** Dot plot of size *vs*. complexity-granularity of sorted granulocytes exposed to fluorescent beads. **(H)** Histograms of the granulocyte sample shown in E: the red histogram indicates the existence of 2–3 granulocyte populations associated to different amounts of fluorescent beads. **(I)** Phagocytosis index of unsorted circulating hemocytes and of sorted hyalinocytes, agranulocytes and granulocytes (means ± SE; N = 6; different letters indicate statistically significant differences, one-way ANOVA, Tukey test). **(J)** Phase contrast micrographs of sorted phagocytic hemocytes. Abbreviations: *agr*, agranulocytes; *gra*, granulocytes; *hya*, hyalinocytes. Scale bar represents 10 μm.

Incubation of fresh hemolymph with fluorescent beads resulted in 24.8% of hemocytes showing phagocytosis and the histograms showed there were 4–5 groups with different amounts of internalized beads (Fig 7A and 7B).

However, when sorted hemocyte populations were exposed to fluorescent beads, all of them showed some phagocytizing ability, though hyalinocytes showed a significantly higher index than those of the other populations (Fig 7I, one-way ANOVA, Tukey test) and which was similar to that of unsorted hemocytes. Hyalinocytes showed 3–4 groups (Fig 7D) with different numbers of internalized beads, while agranulocytes and granulocytes only showed 2–3 groups (Fig 7F and 7H, respectively). Phase contrast microscopy of the sorted phagocytizing hemocytes indicated that hyalinocytes and agranulocytes basically retained their morphological characteristics, but granulocytes were much modified: they became spherical with a degranulated and vacuolated cytoplasm, they frequently emitted long filopodia, and their nuclei were condensed and smaller (Fig 7J).

### Renal hemocyte islets

#### Morphology (HE stain and TEM)

**Fig 8:**
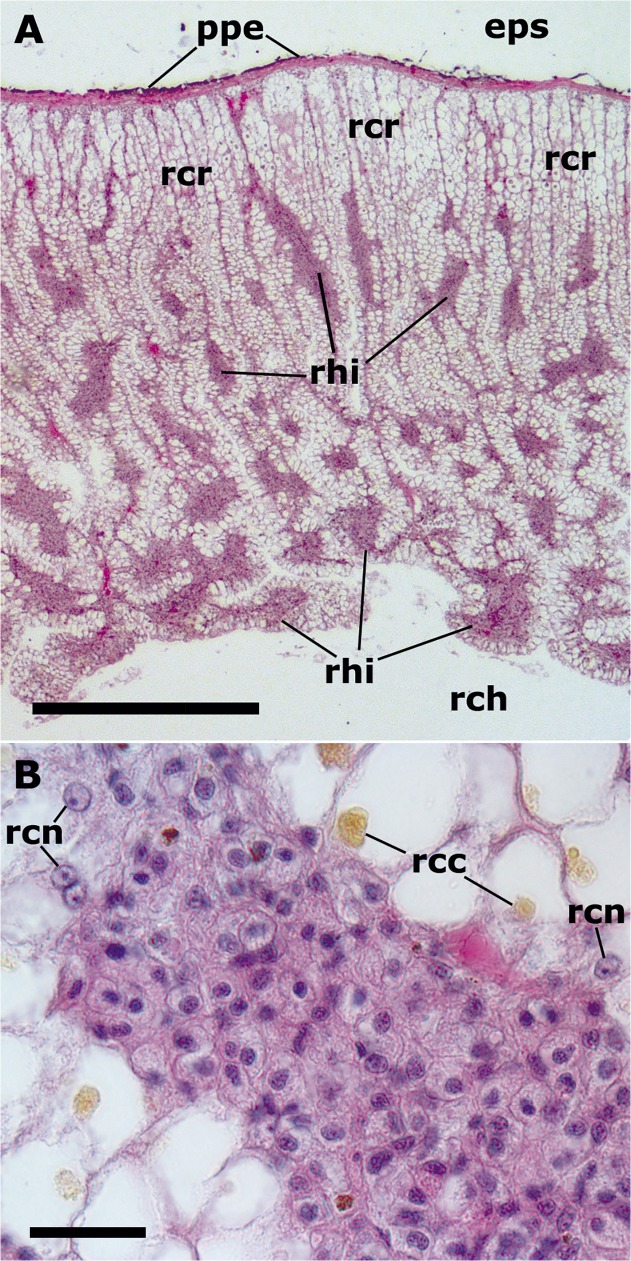
Renal hemocyte islets (HE-stain). **(A)** Kidney section perpendicular to the pallial surface of the organ. The mantle separates the kidney from the extrapallial space and is covered by the pigmented pallial epithelium. Renal hemocyte islets are seen as elongated basophilic masses between the cortical renal crypts, while they appear transversally sectioned in the region overlying the renal chamber. **(B)** Section of a hemocyte islet mostly composed of hyalinocytes and delimited by the renal epithelium. Abbreviations: *eps*, extrapallial space; *ppe*, pigmented pallial epithelium; *rcc*, renal cell concretion; *rch*, renal chamber; *rcn*, renal cell nucleus, *rhi*, renal hemocyte islet.

Under low magnification, the kidney is formed by numerous epithelial crypts perpendicular to external surface of the organ, which lies beneath the mantle (Fig 8A). This organ overlies the renal chamber and the crypts are lined by a cylindrical epithelium whose vesicular cells contain urinary concretions. The hemocyte islets occupy a significant part of the hemocoelic spaces between the crypts and are usually thinner near the mantle surface and more bulky towards the renal chamber (Fig 8A). At higher magnification the islets are hemocyte packs between neighboring renal crypts and are predominantly composed of hemocytes with no eosinophilic granules (Fig 8B).

**Fig 9:**
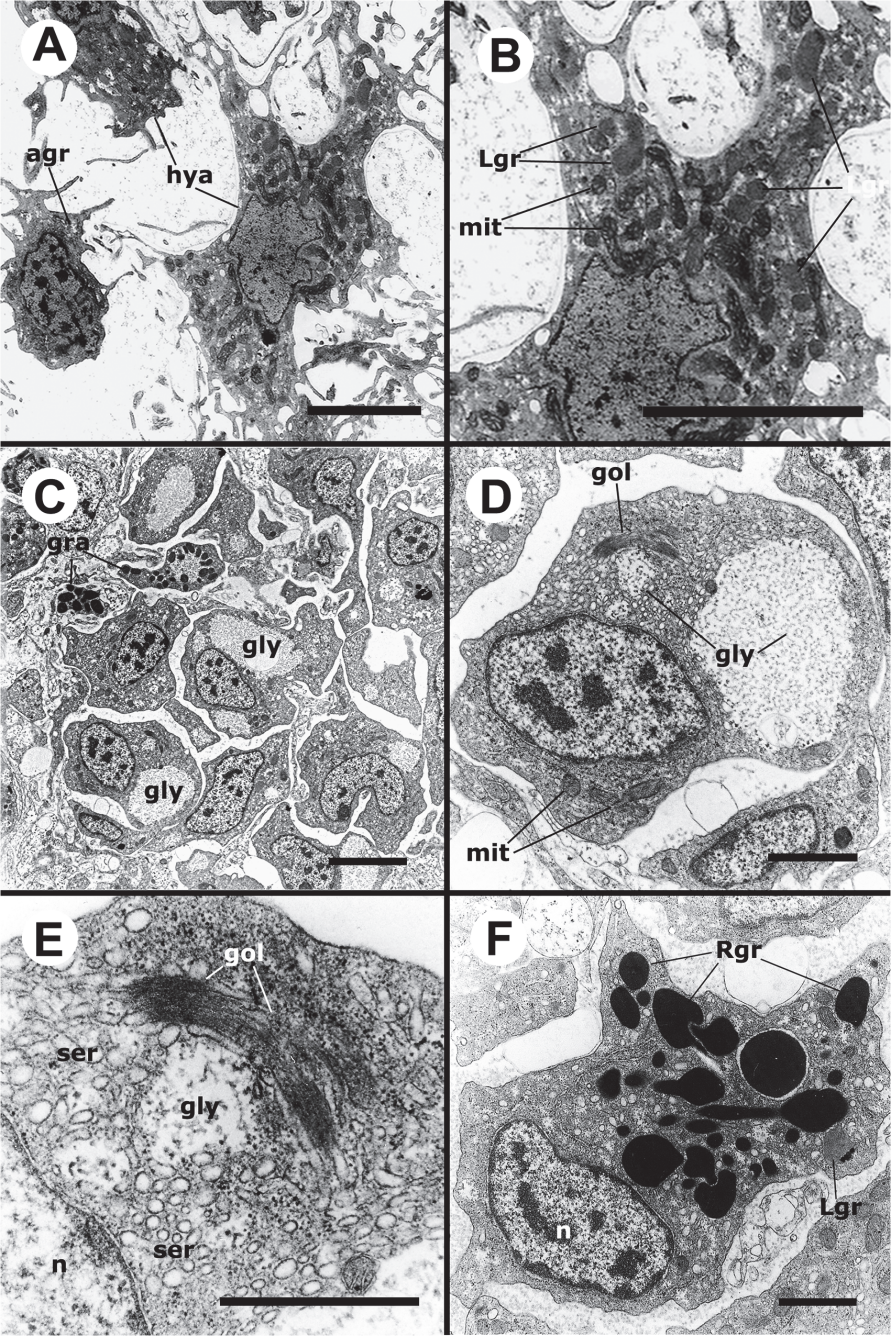
Renal hemocyte islets (TEM). **(A)** An agranulocyte and two hyalinocytes appear loosely attached to the surface region of a renal islet. **(B)** Detail of the larger hyalinocyte on the preceding panel, showing R granules, SER vesicles and numerous mitochondria. The intercellular space contains a microgranular material and membrane remnants. **(C)** Numerous hyalinocytes and two tangential sections of granulocytes at the core of a renal islet. Membrane-unbound areas with presumptive glycogen granules appear in most cells and are larger than those in circulating hemocytes. **(D)** Detail of a cell from the preceding panel, showing a Golgi stack, areas of presumptive glycogen granules, SER vesicles, R granules and mitochondria. The intercellular space is also occupied by a microgranular material and membrane remnants. **(E)** Detail of the Golgi stack and an area of presumptive glycogen granules; numerous SER vesicles and free ribosomes are also seen. **(F)** A granulocyte in a renal islet, showing SER vesicles, some RER profiles and numerous R granules of different sizes (some of them appear merging); a single L granule is also seen. Again, a microgranular material and membrane remnants are found in the intercellular space. Abbreviations: *agr*, agranulocyte; *gly*, presumptive glycogen granules; *gol*, Golgi stack; *gra*, granulocyte; *hya*, hyalinocyte; *Lgr*, L granule; *Rgr*, R granule. Scale bars in A and B panels represent 5 μm, while those in other panels represent 1 μm.

Under TEM, most of the outer surface of hemocyte islets seems exposed to the hemolymph flowing in the hemocoelic spaces that surround them. These superficial hemocytes are more loosely packed than those at the core, and they emit long pseudopodia (Fig 9A) that may participate in trapping foreign particles from the surrounding hemolymph. Less frequently, however, hemocytes appear lying close to the basal membrane of the renal epithelium and even some interdigitations of epithelial cells and hemocytes occur (Fig 9B and 9C). So it is possible that hemocyte islets do not freely float in the hemocoelic spaces but are anchored in some places to the renal epithelium or to the connective tissue underlying it.

Hyalinocytes are predominant in the islets and their ultrastructure is similar to those in the circulation (Fig 9B–9D), except that the membrane unbound areas (presumptive glycogen stores) are larger and more abundant, particularly in those hemocytes in the islet core (Fig 9C–9E) and the Golgi complex is more developed (Fig 9E). Granulocytes and agranulocytes may be occasionally found in islets and are similar to those in the circulation (Fig 9A and 9F). Even though hemocytes appear tightly packed in light microscopy sections of the kidney (Fig 8B), an intercellular labyrinthic space surrounds individual cells in the core of the islet (Fig 9C, 9D and 9F). Numerous pseudopodia are intermingled in this space, which probably hold the hemocytes together and maintain the architecture of the islet. Membrane remnants and a microgranular material are also seen in the intercellular space (Fig 9D and 9F).

#### *In vivo* microbial phagocytosis by kidney hemocytes under TEM

**Fig 10:**
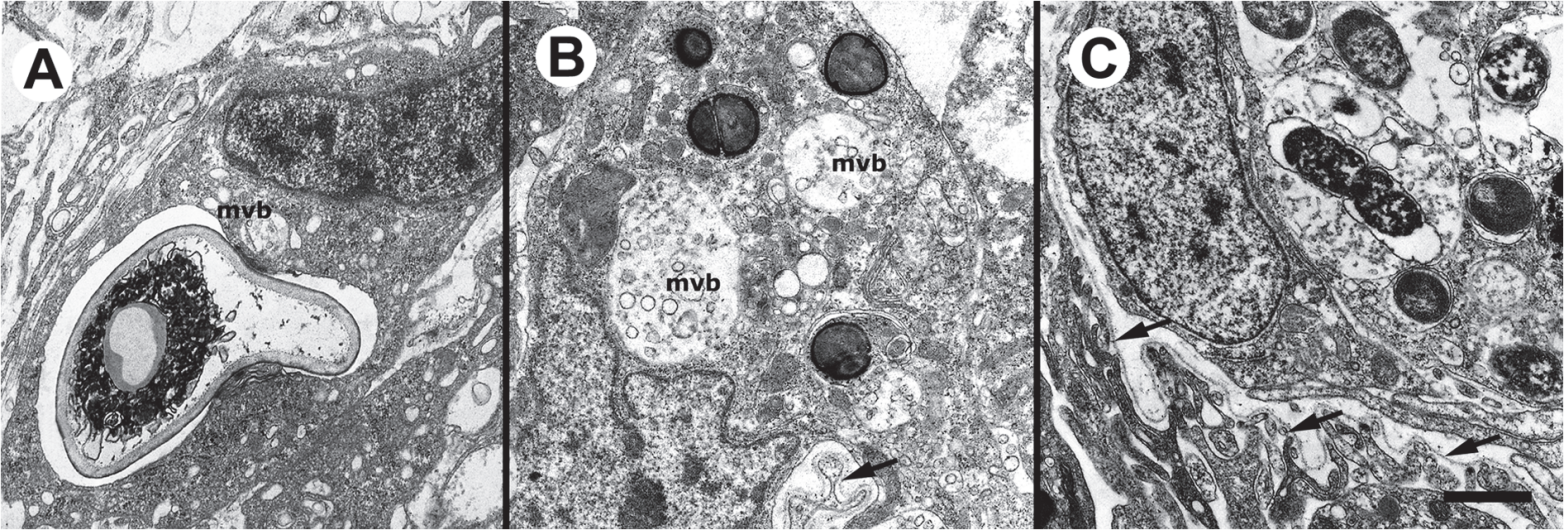
*In vivo* microbial phagocytosis by renal hemocytes (TEM). **(A)** Partly digested yeast cell engulfed by a phagocytic hyalinocyte; a small multivesicular body is seen close to the phagosome; **(B)** Several *S*. *aureus* cells engulfed by a hyalinocyte within individual phagosomes; multivesicular bodies of different sizes are also seen; arrow indicates a basal interdigitation of a renal epithelial cell, surrounded by the basal membrane (arrow); **(C)** *E*. *coli* cells in different stages of digestion are contained within complex phagosomes; the basal membrane of the renal epithelium is indicated by arrows. Abbreviation: *mvb*, multivesicular body. Scale bar represents 1 μm.

Phagocytosis by these hemocytes occurs after injection of microorganisms in the foot. The phagosomes formed are similar to those seen after in vitro phagocytosis, i.e., large phagosomes containing *S. cerevisiae* cells (Fig 10A), small and usually separate phagosomes containing *S. aureus* cells (Fig 10B) and complex phagosomes with several compartments containing *E. coli* cells (Fig 10C). Multivesicular bodies are frequently seen (Fig 10A and 10B).

#### *In vitro* phagocytosis of fluorescent beads by renal hemocytes

**Fig 11:**
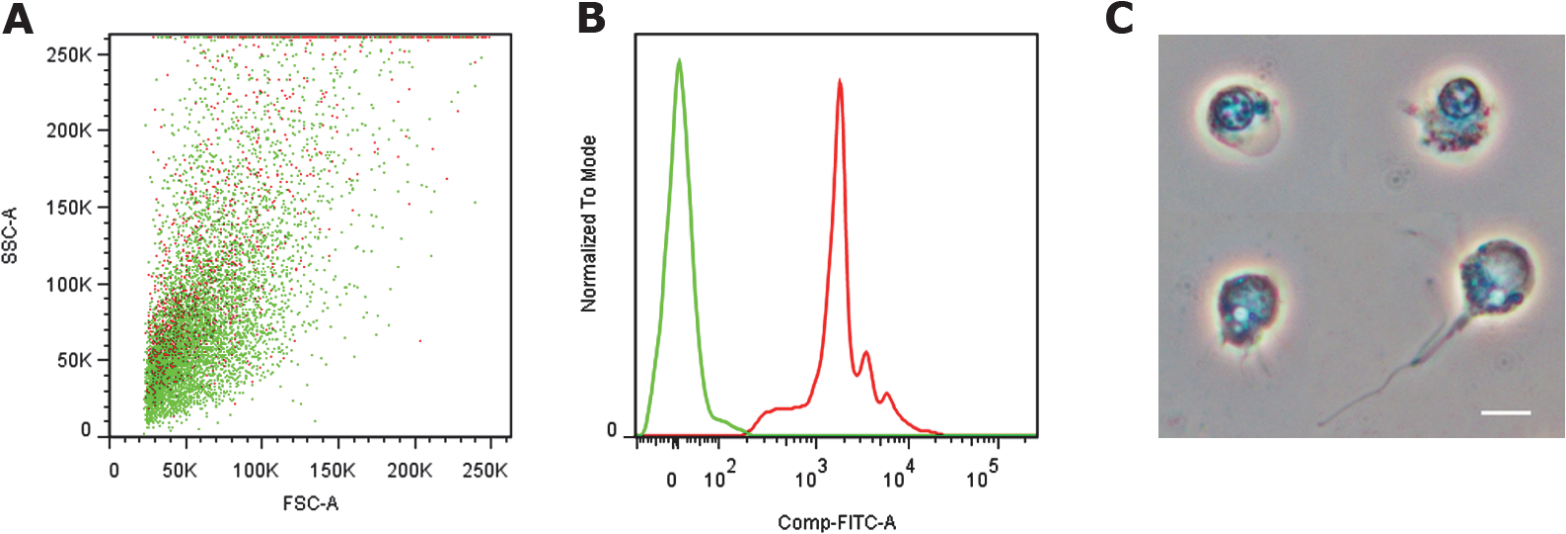
*In vitro* phagocytosis of fluorescent beads by dispersed renal hemocytes. **(A)** Dot plot of cell size *vs*. complexity-granularity of dispersed renal hemocytes exposed to fluorescent beads. Red dots indicate phagocytic hemocytes associated to fluorescent beads, while green dots indicate non phagocytic ones. **(B)** Histograms of the sample shown in A: the red and green lines show the distribution of phagocytic and non-phagocytic hemocytes, respectively. The red histogram indicates the existence of 3–4 hemocyte populations associated to different amounts of fluorescent beads. **(C)** Phase contrast micrographs of sorted hemocytes exposed to fluorescent beads: those in the upper row are not phagocytic while those in the lower row show internalized bead/s. Scale bar represents 10 μm.

Dispersed hemocytes were obtained after collagen digestion of the kidney and they were separated from epithelial renal cells and urinary concretions (S1 Fig). These hemocytes showed a continuum of size and complexity-granularity (Fig 11A). Attempts for further sorting of hemocyte subpopulations were unsuccessful since cell destruction and aggregation occurred. Therefore, the entire hemocyte population obtained was exposed to fluorescent beads and the normalized phagocytic index was 19.3%, i.e., similar to that of circulating hyalinocytes. Also similarly, the histograms showed 3–4 groups internalizing different amounts of fluorescent beads (Fig 11B). Under phase contrast, most these renal hemocytes are similar to circulating hyalinocytes. Fig 11C shows two of them with no internalized beads (upper row) and two that had internalized fluorescent bead/s (lower row).

## Discussion

### Circulating hemocytes and their changes after *in vitro* phagocytosis

The properties of the different hemocyte types found in the circulation are summarized in Table 2. Hemocyte populations in which the expected circulating types (hyalinocytes, agranulocytes and granulocytes, [18–20]) were predominant could be separated by flow cytometry and cell sorting of hemolymph withdrawn from the heart. The most prevalent morphs in each of the hemocyte populations could be confirmed after fixation and HE staining and under phase contrast, and their phagocytic activity could be determined. Accorsi *et al*. [19] could only recognize two regions by flow cytometry (regions I and II, probably corresponding to agranulocytes and hyalinocytes), while they could not recognize the granulocyte region (Fig 1B) because of the high background of their hemolymph samples. This high background may have been caused by their hemolymph collection after inducing a defensive behavior in which the snail fully retracts the head-foot mass and expels hemolymph through a hemal pore [38]. Hemolymph thus obtained may be contaminated with the excreta that the snail also expels. In fact, Accorsi *et al*. [19] have observed hemocytes phagocytizing bacteria in their samples, which is indicative of contamination (hemolymph obtained from the heart is free of bacteria in *P*. *canaliculata* [20]).

**Table 2:** Properties of circulating hemocyte types in *Pomacea canaliculata*.

Normal control hemocytes		Hyalinocytes	Agranulocytes	Granulocytes
HE stain	Nucleus/cytoplasm ratio	Low	High	Low
	Nuclear location and shape	Eccentric, variable shape	Central, round	Eccentric, variable shape
	Cytoplasmic staining	Basophilic or chromophobic	Basophilic	Large eosinophilic granules (likely R granules)
	% of total hemocytes	63 ± 4	28 ± 2	9 ± 3
LysoTracker Red	Acidic granules	Small and round (likely L granules)	None	Large and rod-shaped (likely R granules)
TEM	SER and mitochondria	Abundant	Scarce	Scarce
	Golgi complex	Unfrequently found	Not observed	Well developed
	Cytoplasmic granules	L granules	Occasional L granules	Abundant R granules and occasional L granules
	‘Glycogen’ areas	Occasionally seen	None	None
Flow cytometry	Relative size	Large	Small	Large
	Relative complexity/granularity	Low	Low	Large
**Phagocytizing hemocytes**				
LysoTracker Red	Acidic granules	Loss of small round granules	Not determined	R granules tend to fuse and are occasionally retained;
	Phagosomes	Only the inner part of the complex phagosomes is acidic	Not determined	Not determined
TEM	Degranulation	Loss of L granules	Not determined	R granules’ fusion may precede degranulation
	Phagosome morphology	Varies according to the phagocytized microbe	Not determined	Not determined
Flow cytometry	Phagocytic index (%) after cell sorting	20 ± 4	9 ± 2	10 ± 3

Generalized features of circulating control hemocytes were frequent mitochondria and a well developed endoplasmic reticulum, with numerous vesicles of varying sizes. L granules were predominant in hyalinocytes, even though they also occurred in granulocytes, where R granules predominated. L granules were interpreted as lysosomes, which is also supported by the finding of acidic granules of similar size, shape and cytoplasmic distribution when LysoTracker Red labeling was used. However, even though L granules occur in most hyalinocytes under TEM, acidic granules are only found in some of these cells, suggesting that not all L granules are acidic. Neither Shozawa & Suto [18] nor Accorsi *et al*. [19] reported L granules in their electron microscopy observations. However, micrographs from Shozawa & Suto [18] were not clear enough, and the failure of Accorsi *et al*. [19] to observe L granules was probably due to insufficient magnification and/or to degranulation in response to the bacterial contamination of their samples.

‘Electron dense granules’ were also observed by Shozawa & Suto [18] and Accorsi *et al*. [19] and they should correspond to R granules. Granules of similar size, shape and cytoplasmic distribution were intensely labeled with LysoTracker Red in circulating hemocytes in the current study and should also correspond to R granules. These granules appeared merging in some cells (both under TEM and when using LysoTracker Red), a process that was more intense after microbial exposure (see below). Labeling of acidic compartments with neutral red by Accorsi *et al*. [19] also showed large acidic granules compatible with the R ones.

Membrane-unbound areas containing irregular small clumps were also found in circulating hemocytes, and similar areas were previously found in multicellular spheroidal aggregates of hemocytes *in vitro* [20] and they have been interpreted as presumptive glycogen deposits, following the ultrastructural criteria of Revel *et al*. [39] and the histochemical determinations in other gastropods [40, 41]. Similar electron lucent ‘granules’ (in fact, membrane-unbound areas) were also reported by Shozawa & Suto [18] in circulating hemocytes. These presumptive glycogen deposits are larger and more frequent in hemocytes in renal islets and may suggest a role in the circulation and storage of this metabolically valuable molecule.

When circulating phagocytes were exposed to *S*. *aureus*, *E*. *coli* or *S*. *cerevisiae* cells, L granules (and the corresponding small and round granules in LysoTracker Red preparations) disappeared from the cytoplasm, probably indicating either exocytosis or lysosomal/phagosomal fusion. Yeast cells were engulfed singly, but bacterial cells were engulfed either singly or in groups. *S*. *aureus* cells were usually contained within individual phagosomes while *E*. *coli* cells were within complex phagosomes showing several compartments under TEM. In the latter case, only the compartment in close contact with bacterial cells was acidic (i.e., intensely labeled with LysoTracker Red). Structures resembling L granules were sometimes seen attached to phagosomes under TEM (e.g., Fig 6A) which may be indicative of early lysosomal/phagosomal fusion. Some non phagocytizing granulocytes showed gigantic granules of high electron density which were probably the result of R granules’ fusion. It is likely that these merging granules were participating in a kind of ‘compound exocytosis’ [42] resulting in granulocyte degranulation, as it has been shown in bivalves (e.g., [43, 44–47]) and has been related to the release of lysozyme and other hydrolytic enzymes that may kill bacteria, either in the circulation or in tissue. Release of lyzozyme immunoreactive molecules was increased in serum of an heterobranch gastropod after bacterial challenge [48]. Also, the presence of several bioactive peptides has been shown in hemocytes of *Viviparus ater*, an architaenioglossan gastropod [49].

### Significance of hemocytes in renal islets and their *in vivo* and *in vitro* phagocytizing ability

The existence of renal hemocyte islets has passed unnoticed in early studies on *P*. *canaliculata* [28, 50]. Even though isolated hemocytes and/or occasional hemocyte aggregations may be seen in the connective tissue of *P*. *canaliculata*, the renal islets are the only large hemocyte aggregates that are regularly seen in this species (unpublished observations).

The position of the kidney in the circulation of this snail will ensure hemocytes in renal islets to become in contact with microbes or antigenic molecules in the hemolymph drained from the head-foot mass and to a lesser extent from the visceral hump [28] and hence, renal islets may constitute an important immune barrier. *In vivo* phagocytosis of different microorganisms has been shown here after the injection of microbial suspensions in the foot. Also, dispersed renal hemocytes have shown phagocytic activity when exposed *in vitro* to fluorescent beads. Furthermore, spheroidal hemocyte aggregates are formed between the renal crypts after microbial challenge [27] which also support the role of the kidney as an immune barrier.

The lung is another organ that may act as an immune barrier, receiving hemolymph drained from the mantle cavity and the viscera [28]. Both the kidney and the lung may participate in the reaction to *Angiostrongylus cantonensis*, the causative agent of eosinophilic meningitis, a serious zoonotic disease [51] which utilizes the invading *P*. *canaliculata* as the most frequent intermediate host in China, i.e., in the native range of the parasite [52]. Experimental infection with parasitic larvae has been obtained in another ampullariid snail, *Marisa cornuarietis*, and hemocyte aggregates have also been reported in the lung of infected snails [53]. Even though the lung of uninfected *P*. *canaliculata* does not show hemocyte aggregates similar to those in the kidney, it does form hemocyte aggregates after immune challenges [27]. These aspects are important, because the parasite has recently spread to the native range of *Pomacea* species [54, 55].

## Supporting Information

S1 Fig Obtention of dispersed hemocytes from collagenase-treated kidney tissue. **(A)** Dot plot of cell size *vs*. complexity-granularity of dispersed cells and urinary concretions after collagenase digestion. **(B)** Dot plot of cell size *vs*. fluorescence emission (Comp-PI-A); the framed region was sorted and used to test for phagocytic activity. **(C)** Dot plot of cell size *vs*. complexity-granularity of cells contained within the region framed in B; which were predominantly hemocytes (phase contrast microscopy, not shown in the figure).
(TIF)
