# Supplementary figures and images for: Immune Defenses of the Invasive Apple Snail Pomacea canaliculata (Caenogastropoda, Ampullariidae): Phagocytic Hemocytes in the Circulation and the Kidney
Source: PLoS One. 2015 Apr 20;10(4):e0123964. doi: 10.1371/journal.pone.0123964 (PMC4404100; doi:10.1371/journal.pone.0123964)

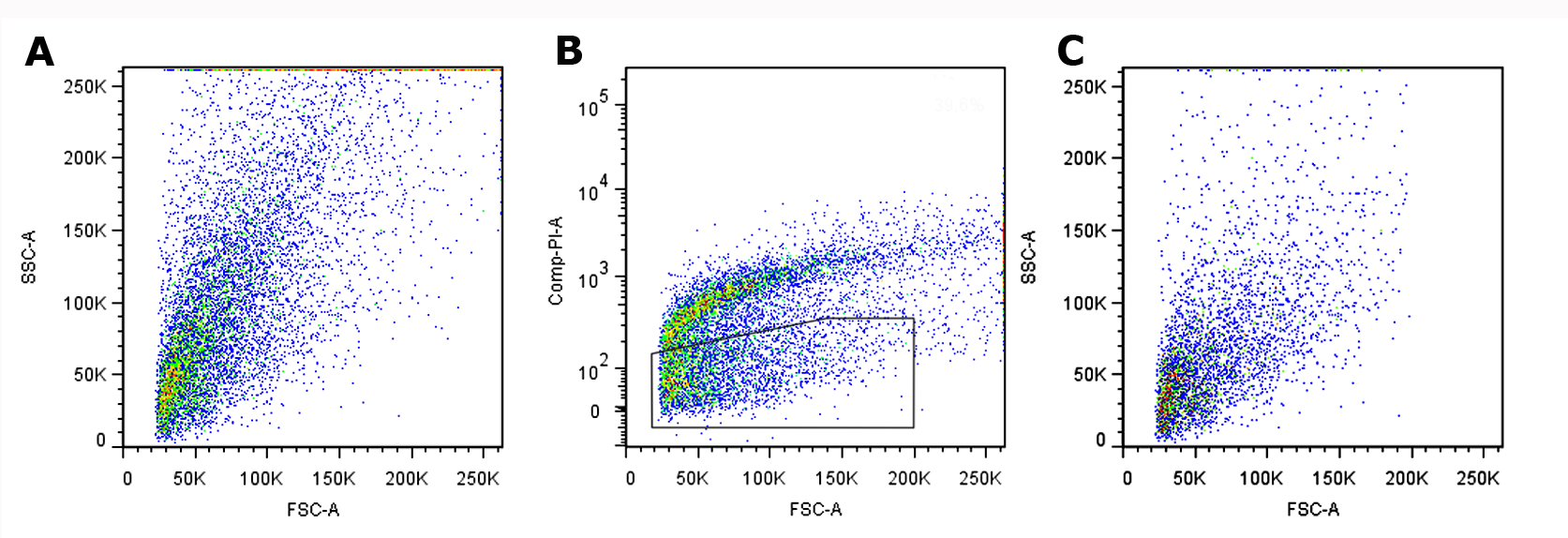

Supplement: S1 Fig — (A) Dot plot of cell size vs. complexity-granularity of dispersed cells and urinary concretions after collagenase digestion. (B) Dot plot of cell size vs. fluorescence emission (Comp-PI-A); the framed region was sorted and used to test for phagocytic activity. (C) Dot plot of cell size vs. complexity-granularity of cells contained within the region framed in B; which were predominantly hemocytes (phase contrast microscopy, not shown in the figure). (TIF) [file pone.0123964.s001.tif]
